# Reactive Oxygen Species Play a Role in the Infection of the Necrotrophic Fungi, *Rhizoctonia solani* in Wheat

**DOI:** 10.1371/journal.pone.0152548

**Published:** 2016-03-31

**Authors:** Rhonda C. Foley, Brendan N. Kidd, James K. Hane, Jonathan P. Anderson, Karam B. Singh

**Affiliations:** 1 CSIRO Agriculture, Centre for Environment and Life Sciences, Floreat, WA, Australia; 2 The UWA Institute of Agriculture, University of Western Australia, Crawley, WA, Australia; Leibniz-Institute of Vegetable and Ornamental Crops, GERMANY

## Abstract

*Rhizoctonia solani* is a nectrotrophic fungal pathogen that causes billions of dollars of damage to agriculture worldwide and infects a broad host range including wheat, rice, potato and legumes. In this study we identify wheat genes that are differentially expressed in response to the *R*. *solani* isolate, AG8, using microarray technology. A significant number of wheat genes identified in this screen were involved in reactive oxygen species (ROS) production and redox regulation. Levels of ROS species were increased in wheat root tissue following *R*. *solani* infection as determined by Nitro Blue Tetrazolium (NBT), 3,3'-diaminobenzidine (DAB) and titanium sulphate measurements. Pathogen/ROS related genes from *R*. *solani* were also tested for expression patterns upon wheat infection. *TmpL*, a *R*. *solani* gene homologous to a gene associated with ROS regulation in *Alternaria brassicicola*, and *OAH*, a *R*. *solani* gene homologous to oxaloacetate acetylhydrolase which has been shown to produce oxalic acid in *Sclerotinia sclerotiorum*, were highly induced in *R*. *solani* when infecting wheat. We speculate that the interplay between the wheat and *R*. *solani* ROS generating proteins may be important for determining the outcome of the wheat/*R*. *solani* interaction.

## Introduction

The soil-borne pathogen, *Rhizoctonia solani* is a damaging necrotrophic fungus causing billions of dollars of losses to agriculture worldwide [1]. This pathogen has a broad host range and infects major crops including barley, canola, legumes, potato, rice and wheat. However, crop losses due to soil-borne pathogens are often underestimated and their economic importance is expected to continue to rise due to the increased implementation of reduced or no-tillage farming practices. *R*. *solani* is classified into fourteen reproductively incompatible anastomosis groups (AGs). One of these, AG8, is a devastating pathogen causing bare patch of cereals, brassicas and legumes [2, 3].

Despite significant efforts in many crops, identification of crop cultivars resistant to *R*. *solani* has proved to be elusive. The wheat Scarlet-Rz1 mutant initially showed promise of resistance against *R*. *solani* AG8 but the enhanced resistance was masked in the field [1]. One of the reasons that resistant cultivars are hard to identify may be because of the necrotrophic lifestyle of *R*. *solani*. Plant pathogenic lifestyles comprise of biotrophs that infect and require living tissue for their growth, necrotrophs that kill and then colonise dead tissue and hemi-biotrophs that have an initial biotrophic phase before switching to a necrotrophic phase. Significant progress has been made in our understanding of plant defence against biotrophic pathogens (reviewed by [4]) in comparison to necrotrophic pathogens. Whilst plants have evolved Resistance (*R*) genes to recognise pathogen encoded effectors and activate changes that leads to a hypersensitive response, it appears that fungal necrotrophs have utilised this recognition mechanism to promote cell-death and therefore pathogen infection and proliferation. Plant R-gene-like recognition genes have been found that recognise specific effectors from necrotrophic pathogens, but these are considered susceptibility genes as the defense response they activate promotes infection by the pathogen [5].

Plants require a different disease management strategy to combat necrotrophic pathogens, and some progress has been made with reports of specific hormones being important including JA, ET, ABA and SA [6, 7]. Reactive oxygen species have also been observed in necrotroph/plant interactions [8, 9] and are important players in basal defense through a mitogen-activated protein kinase (MAPK)- dependent pathway [9–12]. The production of reactive oxygen species (ROS) by the consumption of molecular oxygen during host–pathogen interactions is termed the oxidative burst. The most important ROS are singlet oxygen (^1^O_2_), the hydroxyperoxyl radical (HO_2_·), the superoxide anion O^.-^_2_, hydrogen peroxide (H_2_O_2_), the hydroxyl radical (OH^-^) and the closely related reactive nitrogen species, nitric oxide (NO). The NADPH respiratory burst oxidase homolog (RBOH) proteins are involved in the production of ROS, and show a role in plant resistance to the pathogens *Alternaria brassicicola* [13], *Phytophthora parasitica* [14], *R*. *solani* [15], *Magnaporthe oryzae* [16] and *Botrytis cinerea* [17, 18]. The potential role of ROS in effective defence of Arabidopsis to *R*. *solani* AG8 was also demonstrated by the *dsr1* mutant, which showed that mitochondrial generated ROS is required for resistance to AG8 [19]. Plants can have non-host resistance to specific necrotrophic pathogens and in *Arabidopsis thaliana* this may involve secondary metabolites including tryptophan-derived compounds such as camalexin and glucosinolates [20–22]. In addition, novel disease susceptibility factors for fungal necrotrophic pathogens in Arabidopsis have also been recently identified [23].

ROS production is not only observed in plant responses to disease. Necrotrophic fungi can also regulate intracellular levels of ROS for developmental and virulence. Fungal NADPH oxidases (Nox) are required for pathogenic development and this is consistent with the importance of ROS regulation in the successful pathogenesis of *Sclerotinia sclerotiorum*[24], *Botrytis cinera* [17], *Alternaria alternata* [25] and *Magnaporthe oryzae* [26]. The predicted primary role of Nox in virulence is the generation of spatiotemporal ROS spikes necessary for differentiation of structures such as appressoria and penetration hyphae [27]. In addition, pathogenic fungi have developed specialized, multi-faceted mechanisms to deal with the oxidative stress encountered *in vivo* during infection. The transmembrane protein, TmpL, which is required for infection of plants by the necrotroph *A*. *brassicicola*, is necessary for regulation of intracellular ROS levels and tolerance to external ROS [28]. The Cu/Zn superoxide dismutase (SOD) is involved in inactivating superoxide anions and the SOD gene from *S*. *sclerotiorum* was shown to be important in virulence and induced during plant infection [29].

Recently a number of studies have used gene expression analysis to study specific aspects of *R*. *solani* biology, including during sclerotia formation [30], the response to antagonistic bacteria [31], and during infection of different hosts looking at the plant [15] or pathogen side [32, 33] of the interaction. In this study we have investigated the interaction between wheat and *R*. *solani* AG8 and studied responses from both the plant and pathogen side. We found that a significant number of wheat genes identified in this screen were involved in ROS production and redox regulation. Biochemical staining also confirmed that ROS species were induced during *R*. *solani*/wheat infection. In addition, homologous genes to known ROS/redox related genes were identified from *R*. *solani* and, in some cases, their expression was shown to be affected during plant infection.

## Methods

### Maintenance of *R*. *solani* AG8

The *R*. *solani* isolate AG8 (WAC10335) was obtained from the Department of Agriculture and Food (www.agric.wa.gov.au). *R*. *solani* AG8 was grown on fresh potato-dextrose agar plates (PDA, Sigma-Aldrich, St Louis, MO) at room temperature for 1 week. Millet seeds were used for *R*. *solani* infection and storage. Moist millet seeds were autoclaved and inoculated with *R*. *solani* grown in PDA, at 22°C in the dark for 2 weeks, with occasional mixing. For longer-term storage, inoculated millet seeds were dried overnight in a sterile laminar flow hood and stored at -80°C.

### Wheat growth and *R*. *solani* infection

Two wheat cultivars have been used in this study; Wyalkatchem is a standard wheat cultivar commonly grown in Western Australia [34], and Chinese Spring is the reference cultivar used for the genome sequencing project [35]. *Triticum aestivum* cv Chinese Spring was sourced from Dr. Nicolas Taylor, University of Western Australia, Perth, Australia, and *T*. *aestivum* cv Wyalkatchem was sourced from CSIRO, Black Mountain, Canberra. Plants were grown in a growth cabinet at a temperature range of 21°C to 24°C, with a 16 hour light photoperiod (100 μM.m^-1^.sec-^1^) and were treated with *R*. *solani* AG8 or a mock treatment as described below.

Fine vermiculite was infected by placing four *R*. *solani* infected millet seeds on top of each 5cm x 5cm pot which was covered with foil, contained in a plastic bag for 1 week and left to grow at room temperature. Unsterilised wheat seedlings were germinated on damp blotting paper for one day (Zadoks growth stage GS05) and transferred to pre-colonised pots [36]. After 2, 3 and 4 days, root and shoot tissue from Chinese Spring wheat were collected (3 biological replicates for each tissue/time point; each replicate was taken from a separate individual plant)

For the pathogenicity studies, two-week-old Wyalkatchem and Chinese Spring seedlings were removed from vermiculite and rinsed in water to remove debris and the shoots were weighed for five plants from each cultivar. *R*. *solani* pathogenicity was scored by weighing the shoot mass on both Wyalkatchem and Chinese Spring seedlings with mock and *R*. *solani* treatment, 2 weeks post-infection. Five biological replicates were used for each treatment and Student’s *t*-test was performed on this data.

### Infection conditions for analysis of *R*. *solani* gene expression

Sterile nitrocellulose membrane (Immobilon 82mm, poresize 0.45μm, Millipore, Millipore.com) was placed on the surface of a PDA plate and 1 cm^2^ plug of inoculum from a prior PDA culture of fungus was placed on top of the membrane. The fungus was allowed to grow at 24°C for 1 week until it had almost covered the surface of the 8.5cm diameter nitrocellulose circle. Wheat seeds were surface sterilised in sodium hypochlorite solution (1.7% available chlorine) with shaking for 15 minutes and rinsed 3 times in sterile water. The seeds were added to a petri dish containing filter paper moistened with sterile water and transferred to 4°C for three days, then transferred to 24°C in the dark for 3 days to germinate (Zadoks growth stage GS07). The seedlings were removed from the Petri dishes and added to fresh plates containing 15 mL of minimal medium. The nitrocellulose membrane and attached mycelium was removed from the PDA plate and placed mycelium side down onto the wheat seedlings in minimal medium. The plates were incubated at an average temperature of 24°C for 4 days or 7 days with 16 hours of light (100 μM.m^-1^.sec-^1^) prior to harvesting. The membrane and attached mycelium was removed from the plates, leaving behind the wheat seedlings. The mycelium was peeled from the membrane, blotted dry and frozen in liquid nitrogen. Vegetative fungal samples were treated as above with the omission of wheat seedlings and sampled after 7 days.

### RNA/RT-qPCR/microarray experiments

RNA was extracted from Chinese Spring wheat roots, shoots, and *R*. *solani* AG8 tissue after 4 days and 7 days infection on minimal medium [37] using the Qiagen RNA easy kit following the manufacturer’s protocol (www.qiagen.com). cDNA synthesis and quantitative real-time PCR (RT-qPCR) to measure RNA expression was performed on a Bio-Rad CFX384 icycler based on a previously described method using SYBR green [38] with annealing temperature of 60°C. *R*. *solani* homologous genes were identified by BLAST analysis from the *R*. *solani* AG8 genome sequence [3] using the following sequences from other fungal pathogens; *Epichloe festucae noxA* gene for NADPH oxidase. GenBank: AB236860.1; *E*. *festucae noxB*, *ubc1* genes for NADPH oxidase: Genbank AB236861.1; *Magnaporthe grisea* NADPH oxidase isoform 1 gb|EF667340.1, *M*. *grisea* NADPH oxidase isoform 2, gb|EF667341.1.; *Penicillium marneffei* Cu Zn superoxide dismutase (*sodA*). gb|DQ413185.1; *S*. *sclerotiorum* 1980 superoxide dismutase (SS1G_00699) ref|XM_001598560; *S*. *sclerotiorum* 1980 hypothetical protein, ref|XM_001590428.1; *A*.*brassicicola* transmembrane protein 1 gb|EU223383.1. The *R*. *solani* locus for each identified gene include *noxA* (*RS*AG-8*G_00017*), *noxB* (*RS*AG-8*G_05263*), *noxC* (*RS*AG-8*G_07018*), *noxD* (*RS*AG-8*G_11665*), *TmpL* (*RS*AG-8*G_06411*), *SOD* (*RS*AG-8*G_07316*) and *OAH* (*RS*AG-8*G_03280*). Primers were designed against CDS regions using Primer3 software (http://bioinfo.ut.ee/primer3-0.4.0/primer3/) using default settings with product range between 100-300bp and are listed in S1 Table. The primer efficiencies for each primer pair for the cDNA samples used in this study was measured using LinRegPCR [39] and are listed in S1 Table. As the primer efficiency for all genes was close to the maximum efficiency of 2, the 2^ΔΔCt^ method was used [40]. Wheat EST sequences were extracted from the wheat TGI (The Institute for Genomic Research) database (ftp://occams.dfci.harvard.edu/pub/bio/tgi/data/) including sequences for GLP1 (TC307021), UGE1 (TC424515), TaPR10 (TC427284) and TaGlu (TC368669). ADP-ribosylation factor R has been previously shown to be an effective reference control for wheat genes [41] and thus was used in this study. The *R*. *solani* beta-catenin gene (*RS*AG-8*G_00890*) has been previously used as an internal reference gene for RT-qPCR and was seen to be stably expressed relative to rRNA under wheat infection conditions (data not shown) and thus was used in this study [3]. Statistical comparisons of treatments were performed using a Student’s *t-*test.

One microgram of total RNA from 4-day-old *R*. *solani* infected or mock treated wheat seedlings were sent to AGRF (Australian Genomic Research Facility, www.agrf.org.au) to hybridize using the Agilent Wheat Gene Expression Microarray (www.agilent.com) which contained glass slides formatted with four high-definition 44K probes (60mer oligonucleotides). Three biological replicates of each treatment was included. The content was pre-2008 release; sourced from RefSeq31, Unigene 53, TIGR 11 and TIGR TA5. This Agilent Wheat Oligo microarray with 37,826 probes is registered as GPL13636 in the Gene Expression Omnibus (GEO) at the National Center for Biotechnology Information (NCBI). All expression tag contig sequences used for the design of probes have been published online (KOMUGI, http://www.shigen.nig.ac.jp/wheat/komugi/array/probe/download.jsp).

### Bioinformatic analysis of microarray data

Functional annotations were derived from mapping the probes of the Agilent Wheat Microarray to the latest wheat transcriptome sequences (IWGSC MIPS v2.2) [42, 43]. Probe sequences were mapped to the transcriptome by BLAT (-fastMap) [44] and functional annotations were assigned to probes based on IWGSC MIPS v2.2 InterPro, Pfam and GO functional annotations assigned to transcriptome sequences. Results of this mapping is presented in S1 Dataset.

### ROS detection

Unsterilised one-day-old Wyalkatchem wheat seedlings (Zadoks GS05) germinated on wet filter paper were added to *R*. *solani* AG8 infected vermiculite or mock vermiculite and then grown for a further 3 days. Seedlings were removed from vermiculite and debris was rinsed off the plant. Whole seedlings were treated with 2mM Nitro Blue Tetrazolium (NBT, Sigma-Aldrich) in 20mM phosphate buffer, pH6.1 to detect O_2_^.-^. The seedlings were then rinsed in water to remove excess NBT and photographed using a digital camera (Nikon, D3200).

For H_2_O_2_ detection by 3,3'-diaminobenzidine (DAB) staining, seeds of Chinese Spring and Wyalkatchem wheat were sterilized in 5% hypochlorite, washed 3x in sterile water and placed on sterile filter paper to germinate. Five-day-old seedlings were then transferred to vermiculite that had been pre-treated with *R*. *solani* AG8-infested millet seeds or a mock treatment for seven days at 24°C until harvest. DAB staining was performed using non-acidified 3,3'-diaminobenzidine (Sigma-Aldrich) at a concentration of 1mg/mL, prepared according to the method of Daudi and O’Brien, [45]. AG8-infected and mock-infected wheat roots were gently washed to remove vermiculite and then stained for 30 minutes in DAB solution. Roots were washed in water to remove excess DAB before imaging using a digital camera (Nikon, D3200) oror using a dissecting microscope (Nikon, SMZ-10). Dissecting microscope images were taken at 30x magnification using AmScope software (www.amscope.com).

Biochemical H_2_O_2_ measurement was performed using the titanium sulphate (Sigma-Aldrich) treatment and O_2_^.-^ was measured by NBT. Measurement of NBT reduction, a method used for the determination of O_2_^.-^ was described by Doke [46] and the measurement of H_2_O_2_ by titanium sulphate was originally reported by Snell and Snell [47]. Approximately 100 mg of root tissue, not including 1 cm of root tips, were snap frozen in liquid nitrogen, and ground before suspending in 500 μL of 100 mM phosphate buffer, pH 6.8. The suspension was spun on a table top centrifuge at 14K rpm for 10 min. The homogenate was centrifuged at 19100 g for 5 min at 4°C and the supernatant was used for both H_2_O_2_ and O_2_^.-^ measurement. For the H_2_O_2_ measurement, 160 μl of the supernatant was mixed with 480 μL titanium sulphate solution (0.1% in 20% H_2_SO_4_) and the absorbance measured after 1 min at 410 nm. H_2_O_2_ content was calculated using the extinction coefficient 0.28 l mmol^–1^cm^–1^ and expressed as nmol/gFW [48]. For the O_2_^.-^ measurement, 160 μL of the supernatant were collected and mixed with 480 μL 0.05% NBT. The reaction of the NBT mixture was measured at 405 nm after 5 seconds. There were six biological replicates for each treatment. Statistical comparisons of treatments were performed using a Student’s *t-*test.

## Results

### Identification of wheat genes differentially expressed following *R*. *solani* infection

**Fig 1:**
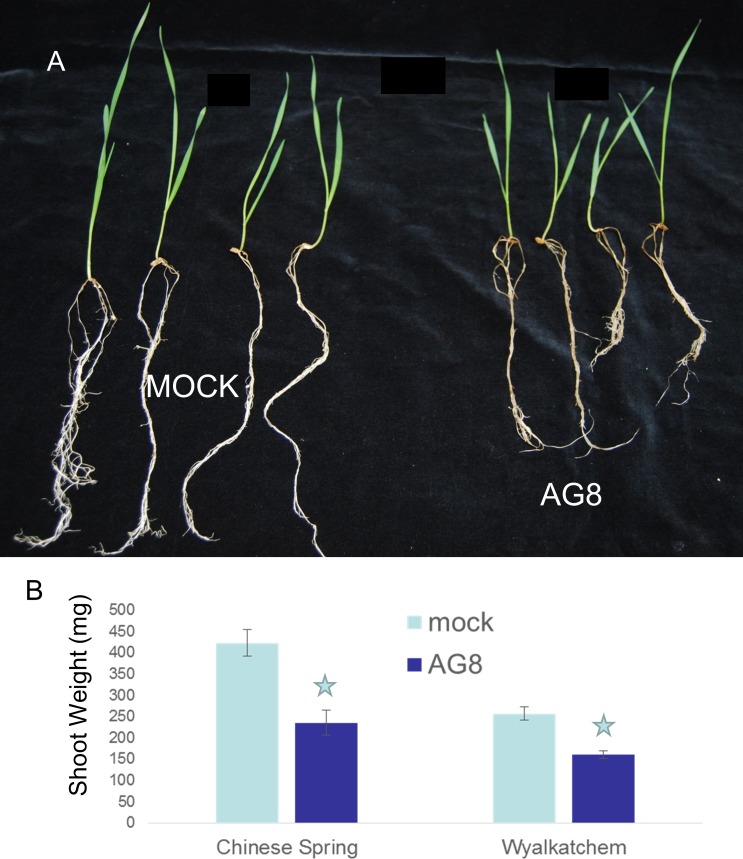
Symptoms of *R*. *solani* AG8 infection of wheat. A) *T*. *aestivum* cv Wyalkatchem seedlings photographed two-week post-infection with AG8 or mock treatment. B) Shoot weight of the wheat cultivars, Chinese Spring and Wyalkatchem following two-week post-infection with AG8 or a mock treatment. Stars indicates significant differences for mock vs *R*. *solani* infection (Student’s *t*-test, *P* <0.05).

A growth cabinet based pathosystem between wheat and *R*. *solani* was modeled on work previously developed for *R*. *solani*/Arabidopsis interactions [49]. By day 4, the pathogenic effect of *R*. *solani* infection on wheat plants could be visualised, with stunted root growth (Fig 1).

RNA extracted from Chinese Spring wheat root tissue from day 4 post-infection with *R*. *solani* AG8 grown on minimal media was used to identify differentially expressed genes compared to mock treatment using DNA microarray analyses. The arrays were obtained from Agilent Technologies, and have 44K, 60 mer probes which have been used to study various aspects of wheat biology [50, 51]. Bioinformatic analysis using a ≥2 fold change cutoff and adjusted p value ≤0.05 identified 83 genes that were induced and one gene that was repressed. A complete set of microarray data from this study was deposited with the GEO repository under the accession number GSE74671, and the differentially expressed genes are listed in S1 Dataset.

The only gene that was identified as being repressed (5-fold and *P* adjusted rate <0.05), was a gene homologous to phospholipase D that has been shown to play a role in regulating lipid metabolism and signalling, which can impact on hormone signalling pathways that mediate plant defence responses and abiotic stresses (reviewed by [52]). Of the 83 genes that were found to be induced (> 2 fold and *P* adjusted rate <0.05), a majority fell into the ROS/redox regulation area or were linked to detoxification, for example five induced genes displayed homology to germin, a gene that catalyzes the conversion of oxalate in the presence of O_2_ into H_2_O_2_ while a number of PR genes were also found to be induced (Table 1, S1 Dataset).

**Table 1:** List of number of wheat genes induced >2 in response to *R*. *solani* and categorised into functional subsets.

Functional Subset	Number of Genes
Detoxifying	15
Flavonoid	9
PR	6
ROS/redox	16
Others	23
Unknown	14

**Fig 2:**
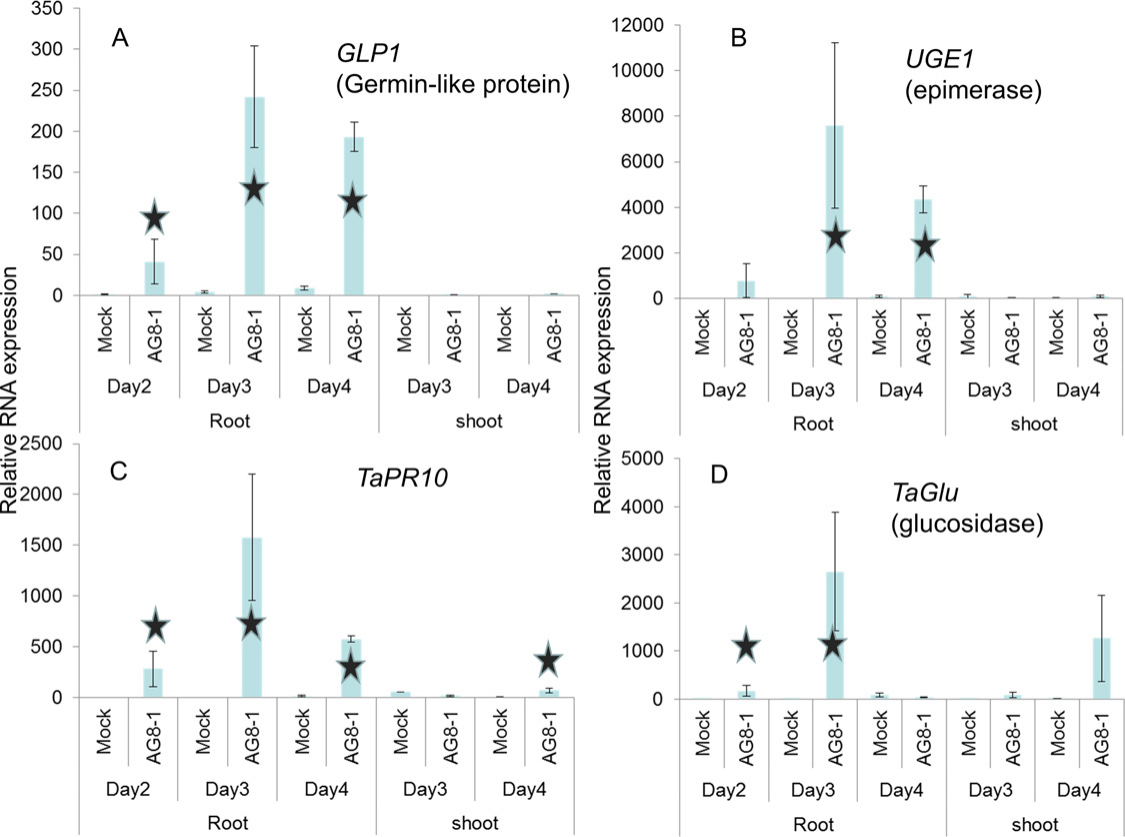
Analysis of selected wheat genes in response to *R*. *solani* AG8 using RT-qPCR. One day old wheat seedlings were infected with *R*. *solani* AG8 for 2, 3 or 4 days, and RNA from root and shoot tissue were tested for relative RNA expression for A) *GLP1* (Germin), B) *UGE1*, C) *PR10* and D) *TaGlu* (glucan endo-1,3-beta-glucosidase GII precursor). RNA expression was normalised to the ADP-ribosylation factor R wheat gene. Stars indicates significant differences for mock vs *R*. *solani* infection (log transformed Student’s *t*-test, *P* <0.05).

To confirm the expression of genes determined by the microarray experiment, four highly induced genes were tested for RNA expression over 2–4 days post-infection with *R*. *solani* AG8 in root and shoot tissue using RT-qPCR (Fig 2). Analysis of root tissue at day 4 following AG8 treatment showed high expression of germin (GLP1), UDP-glucose/galactose 4 epimerase (UGE1) and PR10 verifying the Agilent array data results and they were predominantly expressed in the root compared to the shoot. While *TaGlu* (Beta 1,3-glucanase), was strongly induced at Day 3, there was no significant induction at Day 4 in roots. The *TaGlu* gene sequence was derived from the draft wheat genome sequence but did not produce a perfect match, suggesting other family members in wheat may be contributing to the microarray results.

### The interaction between *R*. *solani* and wheat resulted in the production of ROS

**Fig 3:**
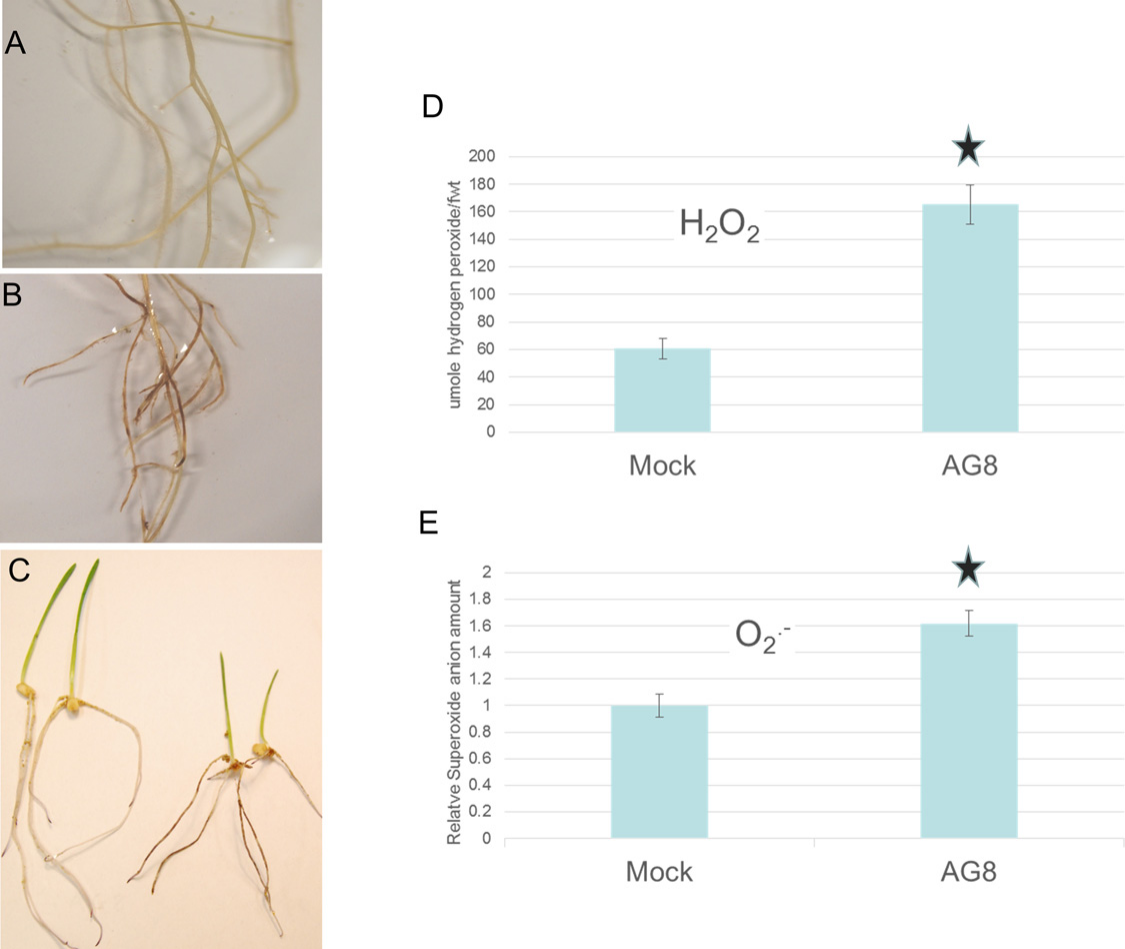
ROS measurement in *R*. *solani* infected wheat. A) Roots of wheat seedlings that were mock infected and stained with DAB, B) Roots of wheat seedlings that were *R*. *solani* AG8 infected and stained with DAB, C) Wheat seedlings infected with mock (left side) or *R*. *solani* (right side) and stained with NBT, D) Biochemical analysis of mock and *R*. *solani* treated wheat roots using titanium sulphate to measure H_2_O_2_. Units are expressed as μmol hydrogen peroxide/fwt and the AG8 treated plants were shown to be significantly different to mock treated (Student’s *t*-test, *P* <0.05) E) Biochemical analysis of mock and *R*. *solani* treated wheat roots using NBT to measure relative superoxide anions per fresh weight. The relative superoxide anion amount was shown to be significantly different to mock treated (Student’s *t-*test, *P* <0.05).

As a number of ROS-related genes were identified from the array, it was of interest to determine the ROS status of the wheat plants during *R*. *solani* infection. Infected seedlings and controls were stained with DAB to measure H_2_O_2_ or NBT to measure O_2_^.-^. In both cases more ROS were seen in the AG8 treated seedling compared to mock (Fig 3A, 3B and 3C). Mock plants had detectable O_2_^.-^ expression in their root tips, and this was extended to the whole root when the seedling was infected by AG8. However, it should be noted that neither of the techniques used to measure ROS could distinguish between ROS produced by the plant and/or the infecting pathogen.

Biochemical analysis also supported the increase of ROS when wheat plants were infected with *R*. *solani* AG8. H_2_O_2_ and O_2_^.-^ were measure by NBT and Titanium sulphate treatment as a colourmetric assay. As shown in Fig 3D and 3E, both H_2_O_2_ and O_2_^.-^ levels are increased by the *R*. *solani* A8-1/wheat interaction at similar induction rates.

### Expression of ROS related genes in *R*. *solani*

**Fig 4:**
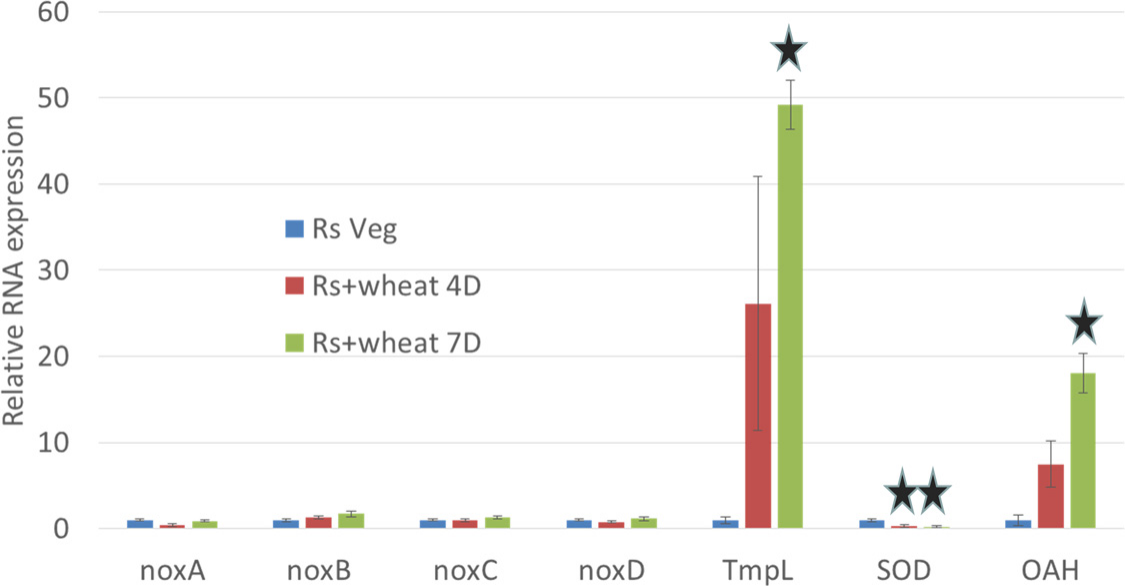
Analysis of *R*. *solani* genes using RT-qPCR in response to wheat infection. Three day wheat seedlings were infected with *R*. *solani* AG8 for 4 or 7 days, and RNA from *R*. *solani* adjacent to the wheat was extracted. Relative RNA expression of *noxA*, *noxB*, *noxC*, *noxD*, *TmpL*, *SOD* and *OAH* was measured. RNA expression was normalised to the *R*. *solani* ribosomal protein gene. Stars indicates significant differences of infection vs vegetative R. solani (Student’s *t*-test, *P* <0.05).

Given the response in wheat to *R*. *solani* AG8 infection involved both the induction of a number of ROS-related genes and increases in ROS, it was of interest to analyse ROS related responses in *R*. *solani*. ROS related genes were identified in *R*. *solani* by nucleotide/protein blast searches from previously characterised fungal genes involved in ROS. Seven *R*. *solani* ROS-related homologous genes were identified from the coding regions of the *R*. *solani* genome [3]; *noxA*, *noxB*, *noxC*, *noxD*, *TmpL*, *SOD* and *OAH*. Fig 4 shows the relative expression of each *R*. *solani* gene following infection in wheat using RT-qPCR. The four *nox* homologous genes did not show any change in expression and the *SOD*-homologous gene was repressed whilst those homologous to *TmpL* and *OAH* showed significant induction compared to *R*. *solani* AG8 growing vegetatively (Fig 4).

## Discussion

*R*. *solani* is a pathogenic fungus that causes physical changes including stunted root growth of the host, transcriptional changes and ROS production. Using differential expression analysis between non-infected and *R*. *solani* infected wheat, 83 genes were identified that were induced and 1 gene that was repressed. Reduced number of differentially expressed genes were detected using the *R*. *solani* AG8 and Chinese Spring wheat Agilent arrays compared to other systems such as the Arabidopsis/*R*. *solani* AG8 (resistant– 7 days post infection) Affymetrix analysis, where 501 genes were identified that were induced and 205 genes were identified that were repressed using similar stringency criteria [15]. In addition, the Arabidopsis/*R*. *solani* AG2-1 (susceptible– 7 days post infection) Affymetrix analysis identified 357 genes that were induced and 229 genes that were repressed [15]. It remains to be determined if there are fewer wheat than Arabidopsis genes affected by *R*. *solani* infection or the wheat probes used in the Agilent array do not accurately reflect the full scope of all wheat genes. It was of interest to note that the genes that were tested for RT-qPCR expression were found to be induced at a higher rate than that seen in the microarrays, suggesting that the microarray may have limitations with regards to sensitivity. Alternatively, because wheat has a hexaploid genome, mRNA expression from other homologs may be masking gene expression changes and therefore RNA sequencing may be required to uncover homolog specific differences in gene expression in response to *R*. *solani*.

Twenty wheat genes were identified from the wheat microarray that were induced greater than ten-fold, and sixteen of these genes show homology to genes with known functions or which have been previously associated with specific conditions such as biotic stress. These included genes homologous to the pathogen-related (PR) genes such as *TaPR5* (thaumatin-like), *TaPR10* and *TaGlu* (glucan endo-1,3-beta-glucosidase GII precursor). The *PR5* and *PR10* genes have been previously shown to be induced during stripe rust infection [53, 54] although the induction was significantly less than what was observed with *R*. *solani* infection. The second most abundant gene was identified as a homolog to UDP-glucose/galactose 4 epimerase (*UGE*), which may have a role in energy production (glycolysis) or inositol production for sequestering phosphate away from bacteria/fungi. Multiple genes from the glucosyltransferase family were identified as being induced during the *R*. *solani* infection. Glucosyltransferase genes have been associated with detoxifying mycotoxins in other fungi Ornithine decarboxylase is required for *Parastagonospora nodorum* virulence in wheat [55], and the fungal pathogen *Fusarium graminearum* may exploit the induction of ornithine as a cue for mycotoxin production during wheat infection [56]. Agmatine coumaroyltraserase has been shown to be the first step of biosynthesis of an antifungal derivative in barley [57]. Ent-copalyl diphosphate synthase functions to synthesise gibberellin/phytoalexin [58] and laccase is an oxidase that has a role in the regulation of lignin production [59].

Two of the genes induced over 10 fold were germin-like proteins (GLP). The plant responds to the toxic effects of oxalic acid by producing the enzyme germin, which catalyzes the conversion of oxalate in the presence of O_2_ into H_2_O_2._ Proteins with 30 to 70% amino acid identities with characterised germin protein sequences are designated as germin-like proteins (GLPs) [60]. The H_2_O_2_ that is released in this reaction may play a role in plant defense as antimicrobial agents, and cross-linking of cells walls or through acting as a messenger in signal transduction pathways linked to oxidative stress signalling (reviewed by [61]). It was of interest to observe that the *R*. *solani OAH* gene was also induced in response to *R*. *solani*/wheat infection. The *S*. *sclerotiorum*–*P*. *vulgaris* interaction also resulted in an induction of an OAH homolog [62]. The *R*. *solani OAH* gene encodes a gene homologous to the enzyme oxaloacetate acetylhydrolase which has been shown to produce oxalic acid [63]. Oxalic acid is believed to act in pathogenesis through acidification of host tissues and sequestration of calcium from host cell walls and has a role in both suppressing and inducing the oxidative burst of the host plant [64–66]. Isolates of *R*. *solani* are known to produce oxalic acid and the virulence of rice infecting strains was correlated with increased oxalic acid production [67]. In addition, over-expression of a germin like oxalate oxidase in rice lead to increased resistance against *R*. *solani* [68]. In our study both wheat *GLP* and *R*. *solani OAH* were induced at both time points used, with no significant difference in expression level between time points. This co-induction of pathogen and host genes with contrasting functions suggests the interplay between wheat GLP and *R*. *solani* OAH may be important in the interaction of *R*. *solani* with wheat.

Wheat and barley germins have been shown to be induced by abiotic stress and biotic stress, although the amount of induction does not necessary correlate with the level of biotic resistance observed [69–71]. In a number of instances, overexpression of germin in Arabidopsis, canola, soybean and tomato can lead to enhanced resistance to fungal pathogens [72–76], however reverse genetic studies of oxalate oxidase in transgenic wheats did not show a role of oxalate oxidase in response to bacterial blight [77].

The enigma of ROS in plant-pathogen interactions, is that pathogens often require ROS for pathogenicity and developing infection structures, and the host requires ROS for resistance. ROS is used by the host and pathogen both directly and indirectly, as for example ROS toxicity is used to kill plant cells or fungal hyphae, while ROS also serves as an important signal molecule. Also, both pathogen and host have detoxifying enzymes to reduce the ROS damage within the organism. The temporal and spatial expression of when, where and by whom ROS is produced and its function during the wheat/*R*. *solani* interaction may hold the key to help understand the respective role(s) ROS plays for the plant and pathogen. Therefore, future research directions are likely to focus on developing experiments to improve resolution and quantification of ROS to address the specific timing and location of production during pathogen infection of plants and to be able to unequivocally distinguish ROS produced by the plant from that produced by the pathogen.

## Supporting Information

S1 Dataset Summary of differentially expressed microarray probes and their functional annotation. (XLSX)

S1 Fig **DAB staining of Chinese Spring (A) and Wyalkatchem (B) roots after AG8 infection**.
(PPTX)

S1 Table Primer sequences used in this study and PCR efficiencies. (DOCX)
