# Supplementary material for: Reactive Oxygen Species Play a Role in the Infection of the Necrotrophic Fungi, Rhizoctonia solani in Wheat
Source: PLoS One. 2016 Mar 31;11(3):e0152548. doi: 10.1371/journal.pone.0152548 (PMC4816451; doi:10.1371/journal.pone.0152548)
Supplement: S1 Fig — DAB staining of Chinese Spring (A) and Wyalkatchem (B) roots after AG8 infection. (PPTX) [file pone.0152548.s002.pptx]

## Slide 1
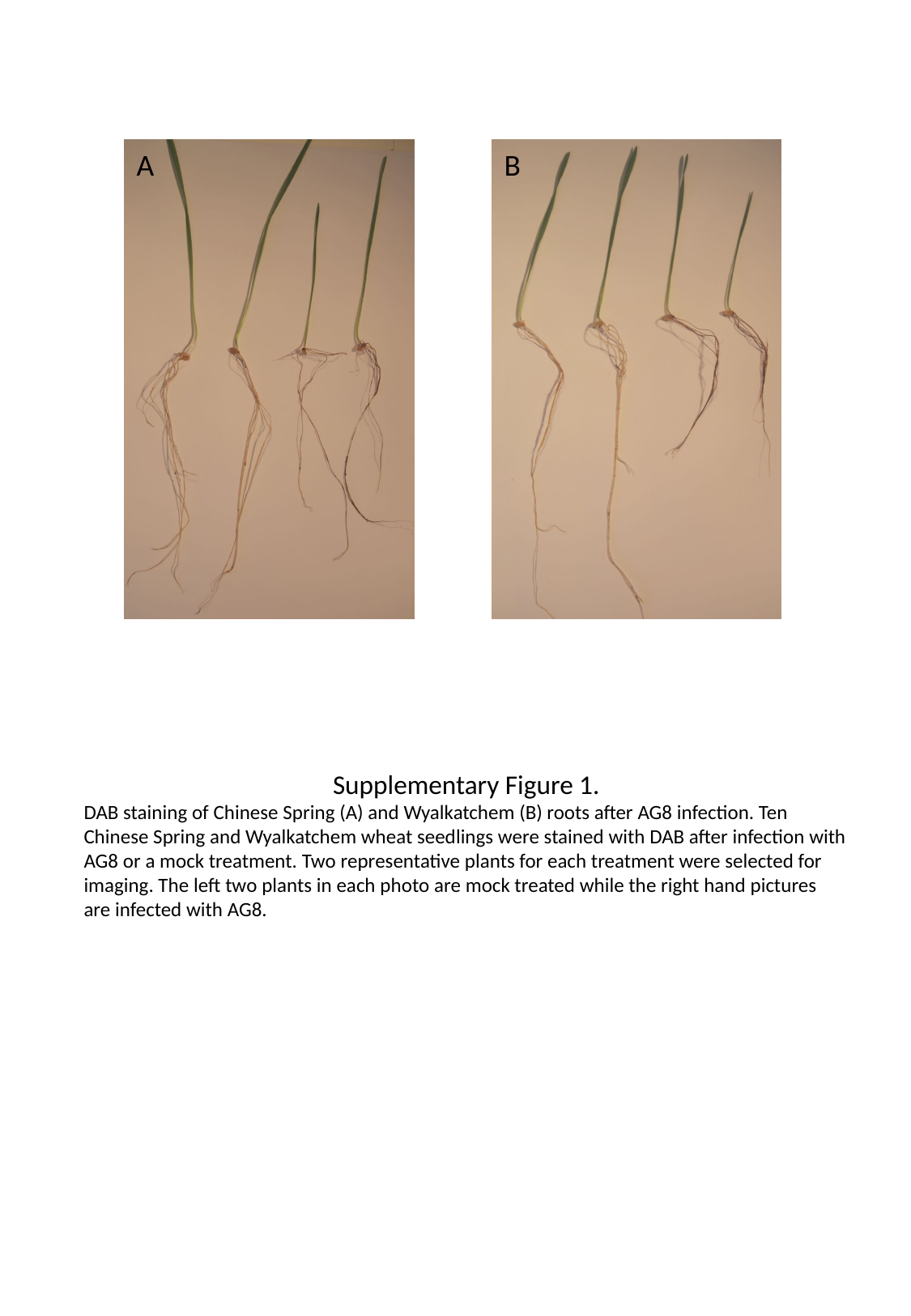

A
B
Supplementary Figure 1.
DAB staining of Chinese Spring (A) and Wyalkatchem (B) roots after AG8 infection. Ten Chinese Spring and Wyalkatchem wheat seedlings were stained with DAB after infection with AG8 or a mock treatment. Two representative plants for each treatment were selected for imaging. The left two plants in each photo are mock treated while the right hand pictures are infected with AG8.
