# Supplementary material for: Reactive Oxygen Species Play a Role in the Infection of the Necrotrophic Fungi, Rhizoctonia solani in Wheat
Source: PLoS One. 2016 Mar 31;11(3):e0152548. doi: 10.1371/journal.pone.0152548 (PMC4816451; doi:10.1371/journal.pone.0152548)
Supplement: S1 Table — (DOCX) [file pone.0152548.s003.docx]

Supplementary Table 1. Primer Sequences Used in this Study and PCR Efficiencies

| **Abbreviation** | **5' primer** | **3' Primer** | **Efficiencies** |
| --- | --- | --- | --- |
| ***ADP*** | GCTCTCCAACAACATTGCCAAC | GCTTCTGCCTGTCACATACGC | 2.19 |
| ***GLP1*** | CCCGATGGAGGTCAATGCGG | CTCAGTGGCGCGAGGGTGCG | 2.06 |
| ***noxA*** | TCAGGCGTCGAACACACG | CAGCCACGAGAGCTTGGG | 2.11 |
| ***noxB*** | CGCATGAGCCACCCAAGG | ACCCCGTTTATCGCTCGC | 2.13 |
| ***noxC*** | ATGGCCACTCTGCGACG | CCGGCTTCCACTCATCCC | 2.11 |
| ***noxD*** | CGACGATCCTCAGGACGC | TCTTGGCTACGTTCGCCC | 2.14 |
| ***OAH*** | TAGCTCGCACTGTCCAAATG | AACGACCATGGGAAGCATAG | 2.13 |
| ***PR10*** | AAGATGGTCGAGGCTTACC | GAAGTCGATCATGAAGCAA | 2.03 |
| ***Ribosomal Prot*** | ATGCTTGGTCTCAAAGCG | ACTTTGATCGCTGTTGCC | 1.99 |
| ***SOD*** | GTGGCCCTGTTTTCATCACT | ACGTTGTCCGAGAAATCCAG | 2.12 |
| ***TaGlu*** | CAGCATAAAGGTTTCCACGTC | GACCTTCATCCCCGGTTT | 1.96 |
| ***TmpL*** | TCCCTACCTATGGCGTCTTG | TTGCTGATGATAGCCACTGC | 2.19 |
| ***UGE1*** | CCGGCAGGTACATCTGCTGC | TCAAAGATCTCACCCAGGTC | 2.01 |
